# Spray-Dried Powder Containing Cannabigerol: A New Extemporaneous Emulgel for Topical Administration

**DOI:** 10.3390/pharmaceutics15122747

**Published:** 2023-12-08

**Authors:** Alice Picco, Lorena Segale, Ivana Miletto, Federica Pollastro, Silvio Aprile, Monica Locatelli, Elia Bari, Maria Luisa Torre, Lorella Giovannelli

**Affiliations:** 1 Department of Pharmaceutical Sciences, University of Piemonte Orientale, Largo Donegani 2, 28100 Novara, Italy; alice.picco@uniupo.it (A.P.); lorena.segale@uniupo.it (L.S.); ivana.miletto@uniupo.it (I.M.); federica.pollastro@uniupo.it (F.P.); silvio.aprile@uniupo.it (S.A.); monica.locatelli@uniupo.it (M.L.); elia.bari@uniupo.it (E.B.); marialuisa.torre@uniupo.it (M.L.T.); 2 APTSol S.R.L., Largo Donegani 2, 28100 Novara, Italy

**Keywords:** cannabigerol, cannabidiol, spray-drying, methyl-β-cyclodextrin, re-dispersible powders, extemporaneous emulgels

## Abstract

Cannabigerol (CBG), a cannabinoid from *Cannabis sativa* L., recently attracted noteworthy attention for its dermatological applications, mainly due to its anti-inflammatory, antioxidant, and antimicrobial effectiveness similar to those of cannabidiol (CBD). In this work, based on results from studies of in vitro permeation through biomimetic membranes performed with CBG and CBD in the presence and in the absence of a randomly substituted methyl-β-cyclodextrin (MβCD), a new CBG extemporaneous emulgel (oil-in-gel emulsion) formulation was developed by spray-drying. The powder (SDE) can be easily reconstituted with purified water, leading to a product with chemical-physical and technological characteristics that are comparable to those of the starting emulgels (E). Thermogravimetric analysis (TGA), attenuated total reflection-Fourier transformed infrared spectroscopy (ATR-FTIR), x-ray powder diffraction (XRPD), and high-performance liquid chromatography (HPLC) analyses demonstrated that the spray-drying treatment did not alter the chemical properties of CBG. This product can represent a metered-dosage form for the localized treatment of cutaneous afflictions such as acne and psoriasis.

## 1. Introduction

The *Cannabis sativa* L. plant contains over a hundred different compounds, including terpenes, carbohydrates, esters, amides, and specific molecules named cannabinoids. Although more than 150 cannabinoids have been isolated from cannabis, the attention has been focused mainly on the “major cannabinoids”: ∆9-tetrahydrocannabinol, cannabinol, cannabidiol (CBD), cannabigerol (CBG), and cannabichromene [1,2,3,4,5,6].

As cannabinoids modulate immune responses and possess anti-inflammatory, antioxidant, anti-aging, and anti-acne properties [7,8,9,10], they have already been proposed for topical (transdermal or local) administration in the treatment of localized skin diseases, including inflammatory, seborrheic, and autoimmune disorders [11,12,13,14,15,16]. Specifically, CBD was effective in treating allergic contact dermatitis, melanoma, psoriasis, and Kaposi sarcoma, exerted an anti-pruritic role in the treatment of atopic dermatitis [17,18,19,20], and potentiated the effect of bacitracin against *Staphylococcus aureus*, *Propionibacterium acnes* (the bacterium responsible for causing acne), and other Gram-positive bacteria [21,22]. CBD also showed antioxidant and anti-inflammatory responses [23,24], and the application of this cannabinoid in inflammatory-based skin diseases has been supported with animal models and pilot clinical studies for the treatment of seborrheic disorders [4]. Similarly, CBG has become highly attractive in dermatology and skin care and has already been proposed for psoriasis and acne. Indeed, CBG showed higher antimicrobial properties compared to CBD and THC against Gram-positive bacteria, mycobacteria, and fungi [25,26,27], and with respect to THC, higher inhibition effects on keratinocyte hyperproliferation, typical for psoriasis [28]. In addition, CBG is efficient in the upregulation of genes involved in wrinkle reduction thanks to the regeneration and proliferation of skin cells and in the upregulation of anti-inflammatory cytokines [25].

When proposed for topical applications, both CBG and CBD have been mainly formulated in the form of oils, emulsions, and semisolid preparations, such as gels and creams. In such formulations, CBD has been associated with hyaluronic acid, boswellic acid, argan oil [29,30,31], *Olea europaea* L. extract, and *Mentha arvensis* L. leaf oil or with a *Calendula officinalis* L. extract [32,33]. The literature also reports examples of CBD in Pickering emulsions stabilized with chitosan/collagen peptide nanoparticles [34] and in microemulgel for the treatment of psoriasis, eczema, pruritus, and inflammatory conditions [35]. There are, instead, limited studies regarding the formulation of CBG for topical application. In the dermatological field, Monou et al. recently developed 3D-printed alginate films containing CBG and CBD, demonstrating their positive effects on the wound healing process [36]; other papers described CBG incorporated in creams with an aloe extract for the treatment of inflammatory skin disorders [37] or in a dermatological oil with CBD for the topical treatment of vitiligo [38]. In the cosmetic field, two recent patents described skin care products containing CBG and CBG/CBD in pomegranate seed oil [39,40].

Although liquid and semisolid dosage forms are widely used for topical application due to their good skin adhesion and spreadability and high acceptance by patients, their shelf life and quality can be challenging due to physical instability phenomena, such as flocculation for creams and emulsions, that can lead to phase separation [41]. In addition, the presence of water in these formulations requires the use of preservatives to avoid the risk of spoilage and to ensure the safety of patients [42,43]. Powders, in comparison, exhibit several advantages: they do not require the massive use of preservatives, possess greater physical and microbiological stability, and have a relatively long shelf life. Furthermore, due to their easy handling, the logistics of powders—storage and transport—are more undemanding and cheaper [44,45]. However, compared to semisolid formulations, powders are difficult to administer topically since they do not adhere to the skin surface.

Considering the constant request for competitive and modern delivery systems even for dermatological applications [46], the purpose of this study was to develop an extemporaneous semisolid preparation as an innovative alternative to overcome the hindrances associated with creams and powders for topical application. Specifically, emulgels (E), oil-in-water emulsions with a gelled external phase [16,47,48], were prepared and spray-dried to obtain re-dispersible powders (SDE) containing CBG. Such powders can be reconstituted as extemporaneous emulgels (E_R) intended for the treatment of cutaneous afflictions. In the preformulation studies, a randomly methylated β-cyclodextrin (MβCD) was selected as a penetration enhancer to prepare, by physical mixing and kneading, binary systems with CBD or CBG that were then characterized by physical-chemical properties, antioxidant activity, and in vitro permeation studies. As CBG was more retained by the skin and thus more suitable for a topical administration with a local effect, it was incorporated into emulgels and then converted into powders by spray-drying. The powders were characterized by the yield of the spray-drying process, appearance, drug content, physical-chemical properties, dynamic angle of repose, and particle size distribution. The starting emulgels and the ones obtained by reconstitution of the spray-dried powders were instead characterized in terms of appearance, pH, drug content, and viscosity.

## 2. Materials and Methods

Non-psychoactive *Cannabis sativa* L., belonging to the IV chemotype, was purchased from Canvasalus Srl (Monselice, Italy). A voucher specimen (Cs-CBG/01/2019) of vegetal material was stored in Novara laboratories. The cannabinoids (CBs), CBD and CBG, were extracted from the fiber hemp in our laboratories. Randomly methylated β-cyclodextrin (MβCD, Cavasol^®^ W7M) and Strat-M^®^ membranes were purchased from Merck KGaA (Darmstadt, Germany). Hemp oil was purchased from ACEF Spa (Piacenza, Italy). A natural powder emulsifier (Instathix^®^ by Alchemy Ingredients, Ascot, Berkshire, UK) composed of a blend of a surfactant, sodium stearoyl lactylate, and three polymeric stabilizers, thickeners, and texture-enhancers (xanthan gum, tapioca starch, and sodium alginate) was kindly provided by Safic-Alcan S.p.A (Milan, Italy). The other solvents and reagents were purchased from Sigma-Aldrich (Milan, Italy); all the chemicals were of analytical grade and were used without further purification. Silica gel 60 (70–230 mesh), reversed-phase (RP) C18 silica gel and Celite^®^ 545 particle size 0.02–0.1 mm, pH 10 (100 g/L, H_2_O, 20 °C), used for low-pressure chromatography and vacuum chromatography, was purchased from Macherey-Nagel (Düren, Germany). TLC silica gel 60 F254 plates 0.25 mm was purchased by Merck KGaA (Darmstadt, Germany).

### 2.1. Cannabinoids Extraction, Isolation and Characterization

**Figure 1:**
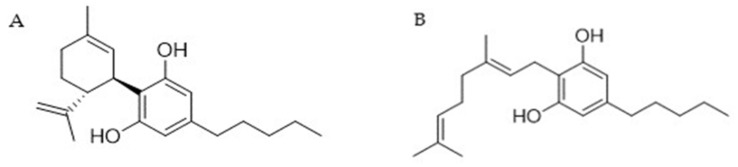
Structure of (**A**) CBD and (**B**) CBG.

Nonwoody *C. sativa* aerial parts (2.290 g), including inflorescences and leaves, were extracted with acetone (2 × 20 L) in a vertical percolator at room temperature, affording 138.74 g (5.7%) of a dark green extract that was dissolved at 45 °C in 700 mL of methanol (1:5 *w*/*v*) and left at 8 °C. After 12 h, the solution was vacuum filtered with cold methanol in a sintered funnel protected by a bed of stratified Celite^®^, obtaining 111.02 g of residual fraction after evaporation with reduced pressure. This latter part was subsequently dissolved in 100 mL of methanol at 45 °C and charged on 300 g of RP C_18_ (1:3 *w*/*w*) in a sintered funnel (18 × 30 cm) and vacuum-filtered with methanol to obtain 101 g of raw residue containing cannabinoid acids. The creamy residue was heated at 130 °C under stirring for 45 min in a paraffin bath to achieve the decarboxylation of cannabinoid acids. The reaction was followed by TLC on silica with a mobile phase composed of petroleum ether and ethyl acetate (80:20 *v*/*v*) and visualized by staining with 5% H_2_SO_4_ in ethanol and heating. This latter decarboxylated fraction was fractionated by low-pressure chromatography (LPC) on silica gel 60 (800 g, petroleum ether-ethyl acetate gradient from 90:10 to 70:30 *v*/*v*) to afford 1.07 g of CBD (Figure 1A) and 8.82 g of CBG (Figure 1B) as a white powder after petroleum ether crystallization. Purification and isolation were monitored by TLC on silica with a mobile phase composed of petroleum ether and ethyl acetate (90:10 *v*/*v*, visualized by staining with 5% H_2_SO_4_ in ethanol and heating). Both CBD (A) and CBG (B) were identified according to ^1^H NMR previously described in the literature [49] (Supplementary Figures S1 and S2). ^1^H 400 MHz NMR spectra were acquired on a Bruker 400 spectrometer (Bruker^®^, Billerica, MA, USA). Chemical shifts were referenced to the residual solvent signal (CDCl_3_: _δH_ = 7.26).

### 2.2. Pre-Formulation Studies

#### 2.2.1. Preparation of and Characterization of CBs-MβCD Binary Systems

CBD and CBG solid–solid interaction with a randomly methylated β-cyclodextrin (MβCD) was investigated, and their binary systems were prepared in a 1:1 *w*/*w* ratio by physical mixing and kneading procedures. Physical mixtures (pm) were obtained by gently mixing CBD and MβCD (pmCBD) and CBG and MβCD (pmCBG) with a spatula for 5 min. Kneaded products (kn), knCBD and knCBG, were prepared by wetting each pm with the minimum volume of methanol and kneading thoroughly with a pestle to obtain a homogeneous slurry [50]. The solvent was entirely removed in a vacuum desiccator for 24 h. All the binary systems were kept in sealed vials at room temperature until analysis.

The residual water content of all the samples was determined as the weight loss between 25 °C and 125 °C by thermogravimetric analysis (TGA 4000 Perkin Elmer, Milan, Italy, 25 ÷ 500 °C, scan rate 10 °C/min, N_2_ 20 mL/min). Furthermore, with the same instrument, the thermal behavior of all the binary systems (pmCBD, pmCBG, knCBD, knCBG) was compared to those of the single components (CBD, CBG, and MβCD), and their experimental parameters, onset temperatures and weight loss percentage were compared to the theoretical ones.

The binary systems were also analyzed through attenuated total reflection-Fourier transformed infrared spectroscopy (ATR-FTIR). The analysis was performed using a Bruker Alpha II instrument equipped with an ATR accessory with monolithic diamond crystal and a DTGS detector, operating in the 4000–450 cm^−1^ range at a resolution of 4 cm^−1^. OPUS Software (Release 8.7) was used for processing ATR-FTIR spectra; the ATR-FTIR spectra of pm and kn systems were compared to those of CBD, CBG and MβCD.

#### 2.2.2. Antioxidant Activity

As CBD and CBG have previously been shown to possess antioxidant properties [23,25], the possible interference of MβCD on their activity was evaluated. The CBs were dissolved in propylene glycol/purified water (80/20 *w*/*w*) and different solutions were prepared as follows: CBD 1% *w*/*w* (CBDsol), CBG 1% *w*/*w* (CBGsol), CBs 1% added of MβCD 1% *w*/*w* (CBD-MβCDsol and CBG-MβCDsol). The DPPH antioxidant assay was performed as previously described in [51] with slight modifications. CB solutions were opportunely diluted in methanol and then left to react with the DPPH radical for 20 min at 25 °C. The antiradical activity was evaluated spectrophotometrically (λ_abs_ = 515 nm) and the final results were expressed as the Trolox equivalent (TE) per gram of sample. MβCD 1% *w*/*w* was also analyzed.

#### 2.2.3. Permeation Study of CBs Solutions

In vitro permeation experiments of the cannabinoid-based solutions (CBDsol, CBGsol, CBD-MβCDsol, CBG-MβCDsol) were carried out using Strat-M^®^ membranes, as already proposed for in vitro permeation studies [52,53]; the experiments were also performed with blank formulations, i.e., without cannabinoids and MβCD. The aim was to select the most suitable cannabinoid, between CBD and CBG, in terms of skin retention, and thus the one more suitable for topical administration with local effect.

The vehicle of the CBs (propylene glycol/purified water-80/20 *w*/*w*) was used as the donor phase according to the study of Casiraghi et al. [54], while the stock solution used as the receptor phase was composed of ethanol/purified water 50/50 *w*/*w* [35]. Both the donor phase and receptor phase were prepared weekly and stored at 4 °C until use. 1 mL of each cannabinoid solution, prepared daily, was loaded into the donor compartment of the diffusion cell. Strat-M^®^ membranes were used immediately with no pretreatment and were placed into the cell with the shiny side facing the donor compartment. The receptor compartment was filled with the receptor phase and after 1, 3, 5, and 24 h, 0.2 mL was withdrawn from the receiver compartment and replaced with fresh medium. The experiments were conducted under occlusive conditions, as the donor compartment was sealed with Parafilm^®^ to avoid sample evaporation, and at the in vivo skin temperature (32 ± 0.5 °C) maintained by circulating water using a thermostatic bath. Each permeation experiment was performed in triplicate.

At the end of the permeation experiments, the Strat-M^®^ membranes were removed from the diffusion cell, gently treated with 10 mL of purified water to wash out the unabsorbed cannabinoids and allowed to dry for 30 min. Then, the tape-stripping technique was used to simulate the removal of the stratum corneum; 35 tape strips were applied consecutively, according to the literature [55]. To extract the cannabinoids from the stripped membranes and to quantify the cannabinoids that were retained, the tapes and the membranes were collected separately in conical tubes, soaked with 10 mL and 30 mL methanol, respectively, and sonicated for 1 h at room temperature. All the extraction liquids were stored at 4 °C in sealed vials until analysis, and the cannabinoids quantification was performed via high-performance liquid chromatography (HPLC, Shimadzu Europe GmbH, Duisburg, Germany) using a Syncronis aQ C18, 5 mm, 150 × 4.6 mm (Thermo Fisher, Monza, MB, Italy) column as stationary phase. The composition of the mobile phase was eluant A: 20 mM sodium phosphate buffer (pH 3.0) and eluant B: methanol. The following gradient at a constant flow rate of 1.0 mL/min was used: from 0 to 10 min, B% from 70 to 90%; from 10.0 to 13.0 min, B = 90%; from 13.0 to 13.5 min, B from 90 to 70%; from 13.5 to 18.5 min B = 70% (total run time 18.5 min). The eluants were filtered through a 0.45 mm PVDF membrane filter prior to use. The sample volume injected was 20 µL, and the detector wavelength was set at 220 nm. Both CBG and CBD eluted at 12.6 min.

After the permeation studies, the distribution of cannabinoids in the “total diffused” and the “in donor phase” fractions was determined and expressed as relative percentages (%, mean values), calculated with respect to the total loaded cannabinoid in the donor compartment. The cumulative recovered amounts, expressed as total “recovery” %, were calculated as the sum of the previously mentioned fractions. Furthermore, the “total diffused” fractions in which the cannabinoids were distributed were expressed as the cumulative amounts of cannabinoids in the receptor compartment (“permeated”), in the water used to wash out the unabsorbed drug (“washed”), in the extraction liquid of the tapes used for stripping stratum corneum (“stripped”), and in the extraction liquid of the membranes (“retained”). The permeation profiles of the cannabinoids were also reported according to the amounts in each aliquot withdrawn at predetermined times, considering the cannabinoids loaded in the donor compartment at the beginning of the experiment.

### 2.3. Formulation Study

#### 2.3.1. Preparation of Emulgels

Semisolid oil-in-gel emulsions, i.e., emulgels (E), can be easily applied to the skin, are non-greasy, and are characterized by a pleasant texture. They contain an oily phase, entrapped in a water-based polysaccharide gel network. Unlike other widely used hydrophilic topical preparations, such as hydrogels, emulgels do not cause skin dehydration, hence, they are particularly suitable for the treatment of skin conditions like xerosis and pathologies such as psoriasis, which are usually associated with dry skin. In this work, two emulgels containing CBG (1% *w*/*w*), one in the absence (Ec) and the other in the presence of MβCD (Ed), were prepared. The water phase and the oily phase were formulated separately. The polymer-based emulsifier (3% *w*/*w*) was premixed into the hemp oil (2% *w*/*w*), along with CBG. Purified water (q.b.) or an aqueous solution of MβCD (1% *w*/*w*) for Ec and Ed, respectively, was heated at 70 °C, directly added to the oily phase, and stirred until homogenous at 3500–5500 RPM for 5 min. Thanks to the thickening and texturizing properties of the stabilizing polymers present in the emulsifier, i.e., xanthan gum and tapioca starch, emulgels were immediately obtained. Ec and Ed, together with the control formulations (Ea without CBG and MβCD, and Eb without CBG), were stored at 4 °C until the spray-drying process. Their composition is reported in Table 1.

#### 2.3.2. Spray-Drying of Emulgels

Emulgels (Ea ÷ Ed) were converted into powders using a spray-dryer (Büchi, Flawil, Switzerland, Mini Spray-Dryer, B-290). The theoretical composition (% *w*/*w*) of their corresponding powders (SDEa ÷ SDEd) is reported in Table 1 and Table 2.

The parameters of the spray-drying process were set based on a previous work performed on aqueous dispersions [56]: nozzle tip diameter 0.7 mm; atomization pressure 2.5 bar; feed rate 12 g/min; inlet temperature 130 °C; outlet temperature 66 °C; aspirator, 100%. Dehumidified air was used as the drying medium to reduce the initial moisture content and relative humidity. 20 g of all the emulgels were spray-dried, in triplicate, and the obtained powders were kept in sealed vials at room temperature until analysis. Due to reasons related to the sustainability of CBG, in this preliminary study, higher quantities (100 g) of only Ea were treated.

#### 2.3.3. Reconstitution of the Spray-Dried Powders

Extemporaneous emulgels were obtained by dispersing SDEa ÷ SDEd with purified water. In a vial, 1 g of SDEa, 1.2 g of SDEb and SDEc, and 1.4 g of SDEd were added with purified water (q.b. 20 g) and homogenized with a spatula for 1 min to obtain Ea_R, Eb_R, Ec_R and Ed_R.

### 2.4. Characterization of the Emulgels

#### 2.4.1. Appearance, pH, Drug Content and Viscosity

A Panasonic Lumix DMC-FZ300 camera was used to visually characterize and evaluate the color and appearance of the emulgels before their drying (Ea ÷ Ed) and of the ones obtained by the reconstitution of the spray-dried powders (Ea_R ÷ Ed_R). The smell of all the emulgels was also checked, and their pH was determined using an LLG-pH Meter 7, LLG Labware, (Meckenheim, Germany).

The viscosity of all emulgels was measured immediately after their preparation (or reconstitution) using a Brookfield DV-II+ Viscometer (small cup adapter, spindle 25; 33 ± 0.5 °C). Each sample was equilibrated for 5 min before measurements to remove the eventual entrapped air bubbles. All the emulgels were analyzed at 20 rpm, and for Ea and Ed, the entire flow curves were also obtained by increasing the rotational speed from 0.05 to 60 rpm (up viscosity ramp) every 2 min; then, the rotational speed was reduced every 2 min from 60 to 0.05 rpm (down viscosity ramp). The temperature uniformity of the sample was ensured by immersing the small cup adapter in a thermostatic jacket. All the shear stress (SS) values obtained from the measurements were recorded and plotted on a graph as a function of the shear rate (SR) values.

#### 2.4.2. Drug Content Determination

The quantitative determination of CBG in the emulgels Ec and Ed was performed by HPLC using the method reported in Section 2.2.3. A CBG standard calibration curve was plotted in the range of 0.1–100 µg/mL.

### 2.5. Characterization of Powders

The powders obtained by spray-drying were characterized in terms of appearance (see Section 2.4.1), drug content (see Section 2.4.2), percentage yield, physical-chemical properties, dynamic angle of repose measurement, and particle size distribution, as detailed below.

#### 2.5.1. Spray-Drying Process Yield

The percentage yield of the drying process was calculated as the ratio between the weight of the powders recovered after spray-drying and the weight of the non-water ingredients used to prepare the emulgels [57].

#### 2.5.2. Physical-Chemical Properties

The spray-dried powders were characterized by thermogravimetric analysis (TGA 4000 Perkin Elmer, 25 ÷ 600 °C, scan rate 10 °C/min, N_2_ 20 mL/min): the residual water content was determined in the 25 ÷ 125 °C range, and their qualitative composition was compared to those of the single ingredients of the emulgels (CBG, MβCD, hemp oil and emulsifier). ATR-IR spectra of SDEa ÷ SDEd and their single components were acquired as reported in Section 2.2.1. The spray-dried powders, CBG, MβCD and the emulsifier were also analyzed by x-ray powder diffraction (XRPD); XRPD patterns were recorded on unoriented ground powders on a Bruker D8 Advance Powder Diffractometer (Karlsruhe, Germany) in reflection mode with Bragg–Brentano geometry, operating with a radiation source of Cu kα monochromatic x-rays (λ = 1.54062 Å). XRPD patterns were recorded in the 8–70° (2θ) range, at voltage and amperage of the source 40 kV/40 mA, at a scan speed of 0.100 s/step, and a step size of 0.01°.

#### 2.5.3. Dynamic Angle of Repose Measurement

The dynamic angle of repose of SDEa ÷ SDEd was determined using a rotating drum (inner diameter 2.5 cm, depth 1.4 cm). The back of the drum was covered with black tape. Each test was conducted using 100 mg of the spray-dried powders, and the rotation frequency of the IKA^®^ RW 16 stirrers was set to 3 Hz (180 rpm). To record the motion of the powders in the drum, the Panasonic Lumix DMC-FZ300 camera was used (1080p HD, 240 frames per second), placed horizontally to the central axis of the rotating drum [58]. The angles between the baselines and the lines traced on the slope of each powder were calculated using the software ImageJ (Version 1.53k), and the powders were classified according to European Pharmacopoeia [59]. The results were reported as the mean value and standard deviation of 5 determinations for each spray-dried powder.

#### 2.5.4. Particle Size Distribution

The particle size distribution of the powders was evaluated by a laser particle size analyzer (Bettersizer 2600, Bettersize Instruments, Munich, Germany) with dry dispersion model equipment (BT-903). A total of 200 mg of each sample, without sieving, was weighed in a sample tube, and the tube was placed into a feeding port. The powder was transported by compressed air (Venturi nozzle) through to the horizontal measuring cell. Results were obtained at 25 °C and 1.52 refractive indexes, collected as mean values and standard deviations (n = 2), and reported as D90 and SPAN values, which represent the 90th percentiles of the cumulative volume distribution and the width of the particle size distribution, respectively [60].

### 2.6. Statistical Analysis

Results were expressed as the mean ± standard deviation (SD) of at least three independent experiments. Differences were estimated by analysis of variance (ANOVA) followed by Tukey’s honest significant difference test. The statistical significance level was set at 0.05. All statistical analyses were performed using the free statistical software R version 4.2.3.

## 3. Results and Discussion

### 3.1. CBs-MβCD Solid–Solid Interaction

**Figure 2:**
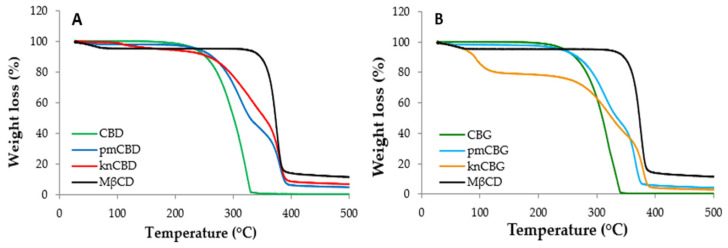
Thermogravimetric profiles of (**A**) CBD, pmCBD, knCBD, and MβCD and (**B**) CBG, pmCBD, knCBG, and MβCD.

The TGA results of the solid–solid interaction study between cannabinoids and MβCD are reported in Figure 2 and Table 3. CBD and CBG show a comparable similar degradation profile, with a single step starting at 140.8 °C and 135.7 °C, respectively (Table 3). MβCD presents a first weight loss (4.4%) in the 25 ÷ 125 °C temperature range (Figure 2), related to the loss of physisorbed water, and a second step (>310 °C) attributable to the degradation of the oligosaccharide structure of the cyclodextrin [61,62]. The thermal degradation of the physical mixtures (pm) and kneaded systems (kn) takes place in three steps: the first is related to solvent loss and the others to cannabinoid and MβCD decomposition. The weight loss of the binary systems pmCBD, pmCBG and knCBD in the 25 ÷ 125 °C range is attributable to the moisture content and water percentages of 2.2%, 1.7% and 3.0%, respectively. knCBG shows a higher solvent content (19.4%) ascribable to the incomplete evaporation, during the drying of the product, of the methanol used in the kneading procedure. Indeed, this kneaded system appeared sticky and with a honey-like consistency. The onset temperature (T onset) degradation of CBD in the presence of the MβCD (pmCBD) shifts marginally at higher temperatures in comparison with CBD alone (143.4 °C vs. 140.8 °C); in contrast, for knCBD, the shift is more accentuated (186.6 °C vs. 140.8 °C). The degradation of pmCBG and knCBG starts at 152.4 °C and 170.7 °C, respectively, more than 16 °C and 35 °C higher compared to CBG alone (Table 3). Considering the T onset observed for pm and kn samples, it is possible to hypothesize a thermal stabilization of the cannabinoids by the MβCD.

The experimental weight loss percentages of the cannabinoids in the pm and kn samples in the temperature range 125 ÷ 500 °C are reported in Table 3 compared to the theoretical ones. Both the pmCBD (53%) and the pmCBG (51%) values are very close to the theoretical ones (50%), while for knCBD (40.8%) and knCBG (40.2%), the weight loss percentages are lower than theoretical ones (50%). These results confirm that a partial cannabinoid thermal stabilization occurred in the presence of the MβCD in the kneaded products; however, the cannabinoids-MβCD interaction was just partial since thermal events related to free cannabinoid molecules were still present in the considered temperature range.

**Figure 3:**
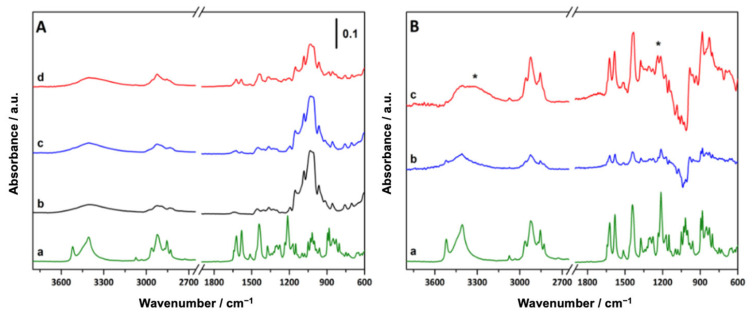
(**A**) ATR-FTIR spectra of CBD (a), MβCD (b), pmCBD (c), and knCBD (d). (**B**) CBD ATR-FTIR spectrum (a) and different spectra obtained after subtracting the MβCD spectrum from the pmCBD spectrum (b) or knCBD spectrum (c). The most significant modifications of the CBD spectrum are marked with asterisks (*).

**Figure 4:**
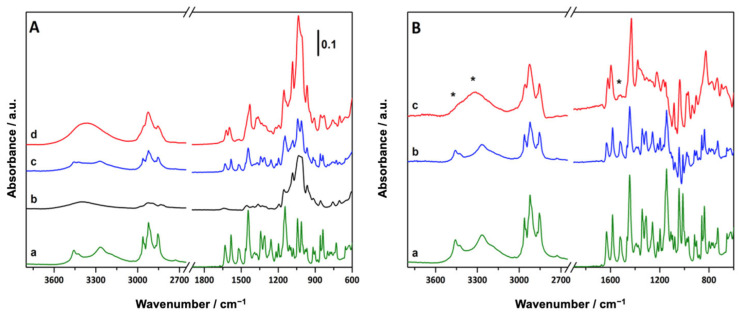
ATR-FTIR spectra of (**A**) CBG (a), MβCD (b), of pmCBG (c), and knCBG (d) and (**B**) CBG (a) and different spectra obtained after subtracting the MβCD spectrum from the pmCBG spectrum (b) or knCBG spectrum (c). The most significant modifications of the CBG spectrum are marked with asterisks (*).

The binary systems, cannabinoids–MβCDs, both pm and kn, were characterized by ATR-FTIR spectroscopy to monitor possible modifications in the shape, intensity, and position of characteristic peaks as a consequence of the different possible interactions between the components. The ATR-FTIR spectra of the pure CBs and MβCD, as well as of the binary systems, are reported in Figure 3 and Figure 4. In the ATR-FTIR spectrum of CBD (Figure 3A, curve a), a complex set of signals can be identified, the most relevant assigned as follows: the signals at 3519 cm^−1^ and 3406 cm^−1^ are due to the stretching vibration of OH groups while the bands at 3073 cm^−1^ and 3032 cm^−1^ are ascribable to the C-H stretching vibrations of aromatic as well as alkene protons. Symmetric and asymmetric stretching of methyl and methylene groups are responsible for the signals at 2962 cm^−1^ (ν_as_CH_2_), 2923 cm^−1^ (ν_as_CH_3_), 2871 cm^−1^ (ν_s_CH_2_) and 2828 cm^−1^ (ν_s_CH_3_). In the low-frequency region, benzene skeleton vibrations are responsible for the signals at 1622 cm^−1^, 1578 cm^−1^, 1510 cm^−1^ and 1441 cm^−1^, while signals at 1375 cm^−1^ and 1212 cm^−1^ can be assigned to the bending modes of methylene groups and C-O stretching vibration, respectively [63]. A similar set of signals can be identified and assigned in the ATR-FTIR spectrum of CBG (Figure 4A, curve a), where the high-frequency region is dominated by O-H (3460 cm^−1^ and 3262 cm^−1^) and C-H (Ar-H at 3032 cm^−1^, ν_as_CH_2_ at 2962 cm^−1^, ν_as_CH_3_ at 2922 cm^−1^, ν_s_CH_2_ at 2871 cm^−1^ and ν_s_CH_3_ at 2855 cm^−1^) stretching vibrations. Similarly to CBD, in the low-frequency region of the spectrum of CBG, benzene skeleton vibration signals can be identified between 1650 and 1400 cm^−1^; signals due to methyl and methylene group deformation and C-O stretching vibration giving rise to bands in the 1400–1000 cm^−1^ range.

The spectrum of MβCD (curve b in both Figure 3A and Figure 4A) is characterized, in the high-frequency region, by a broad band between 3700 and 3000 cm^−1^ (centered at 3402 cm^−1^) assigned to the stretching vibrations of OH groups (as such and H-bonded) and by signals in the 3000–2800 cm^−1^ range, due to the stretching vibration of the C-H bond of the cyclodextrin ring and methyl groups. In the low-frequency region, the presence of water is highlighted by the peak of HOH bending at 1645 cm^−1^ and signals ascribable to C-O and C-O-C stretching vibrations are visible between 1200 and 1000 cm^−1^ [61,62]. In the ATR-FTIR spectra of the pm (curve c, Figure 3A and Figure 4A) and kn systems (curves d, Figure 3A and Figure 4A), both the signals of the cannabinoids and MβCD are visible. Nevertheless, the broad features of MβCD tend to partially mask the signals of the cannabinoids, especially in the O-H stretching region and below 1200 cm^−1^. For this reason, the FTIR spectrum of MβCD has been subtracted from the spectra of the pm and kn systems to better illustrate the presence and eventual modification of the cannabinoid signals, which can infer interactions between MβCD and CBD or CBG; different spectra are reported in Figure 3B and Figure 4B. Cannabinoid signals are still visible after the physical mixing or kneading with MβCD even though some modifications occur: in the low-frequency region, a decrease in the relative intensity of the band at about 1200 cm^−1^ assigned to C-O stretching vibration is evidenced. Additionally, the O-H stretching vibration bands in the high-frequency region of both CBD and CBG are strongly reduced and broadened after both the physical mixing and kneading procedures. This evidence suggests that the OH groups of both CBD and CBG are partially involved in interactions with MβCD and that these interactions are stronger in the kn systems. Nevertheless, the residual presence of C=C stretching vibration signals indicates that only partial interaction occurs, without the formation of inclusion complexes, where the association of the double bond moieties of cannabinoids with the inner part of MβCD would lead to the disappearance of the corresponding signals in the FTIR spectra [64,65].

These results, together with those obtained by TGA, demonstrate that a modest interaction occurred between the cannabinoids and MβCD in the case of the pm and kn systems. However, the complete inclusion of the cannabinoid molecules in the cyclodextrin cavity did not occur and the observed interaction is most likely attributable to the solvent used during the kneading process.

### 3.2. Antioxidant Activity

The DPPH radical scavenging activity of CBD and CBG in the presence of MβCD (both 1% *w*/*w*, in propylene glycol/purified water 80/20 *w*/*w*) was 23.7 ± 1.5 vs. 27.6 ± 1.2 mg TE/g of sample, respectively. Cannabinoids alone possessed antioxidant activity accounting for 25.7 ± 1.3 mg TE/g and 28.4 ± 1.2 mg TE/g for CBD and CBG, respectively, while MβCD showed no activity. CBG was the cannabinoid with the highest antioxidant activity (*p* < 0.01); furthermore, differently from what was observed by Li et al. for the β-cyclodextrin and 2,6-di-O-methyl-β-cyclodextrin [66], MβCD did not influence the antioxidant activity of CBD nor CBG, and no significantly different values with or without it were observed (*p* > 0.05).

### 3.3. Permeation Results

The dermal and transdermal absorption of cannabinoids was evaluated through ex vivo and in vitro and permeation studies using the human epidermis [54], an artificial skin membrane (Skin-PAMPA^TM^) [35], porcine skin [34], or human skin membranes [67]. However, none evaluated cannabinoid permeation through skin biomimetic membranes, such as Strat-M^®^, that share a similar structure with the human skin since they are composed of multiple layers, including a tight outermost one that mimics the stratum corneum of the human epidermis.

In the chromatographic conditions used, CBG and CBD behave similarly: they eluted at 12.7 and 12.5 min, respectively. Table 4 reports the results of cannabinoid distribution at the conclusion of the permeation tests as the amount (expressed as relative percentage) in the donor phase, the cumulative “total diffused” amounts in the receptor phase, and the fractions “retained”, “washed”, “stripped” and “permeated” in which the cannabinoids were distributed. The “recovery” %, calculated considering the mass balance of cannabinoids among the donor and receptor compartments, is approximately 70% for CBDsol and CBD-MβCDsol, and about 77% and 83% for CBGsol and CBG-MβCDsol, respectively, compatible with in vitro experiments conducted with Franz cells.

**Figure 5:**
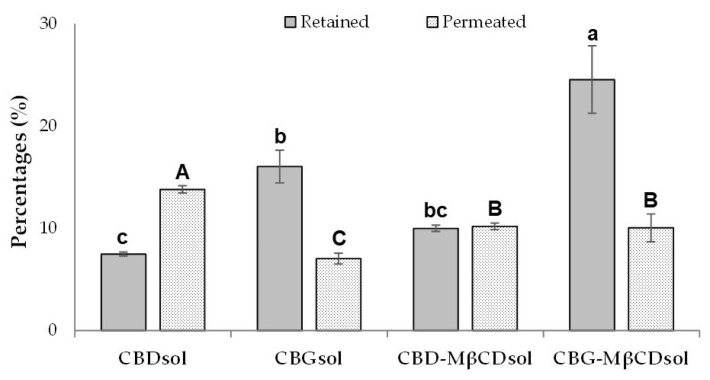
“Retained” and “permeated” percentages of CBs calculated on the “total diffused” amounts from the cannabinoid-based solutions. Different lowercase letters correspond to significant differences between the “retained” percentages, while different uppercase letters highlight significant differences between the ”permeated” percentages.

As regards the distribution of cannabinoids in the different compartments, calculated with reference to the “total diffused” amounts, it can be observed that the percentages of “washed” and “stripped” are very low. The “retained” and “permeated” cumulative amounts of CBs calculated on the “total diffused” amounts from the cannabinoids-based solutions are graphically represented in Figure 5.

Clearly, CBD and CBG permeated the Strat-M^®^ membrane differently. In the case of CBDsol, higher amounts of the drug were found in the receptor phase (“retained”: 7.5 ± 0.2% than “permeated”: 13.8 ± 0.4%), whereas most of the CBG released from the vehicle was retained in the membrane (16.0 ± 1.6% “retained” vs. 7.0 ± 0.5% “permeated”). CBD “permeated” percentages are significantly higher for CBDsol compared to CBD-MβCDsol (13.8 ± 0.4% vs. 10.2 ± 0.3%), while the retention of the drug in the membrane was slightly promoted by the cyclodextrin (7.5 ± 0.2% for CBDsol vs. 10.0 ± 0.3% for CBD-MβCDsol). The drug “permeated” from CBGsol significantly differs from CBG-MβCDsol (7.0 ± 0.5% vs. 10.0 ± 1.4%) and the same behavior can be observed for the “retained” quantities (16.0 ± 1.6% vs. 24.5 ± 3.3%). It is noteworthy that CBG “retained” was significantly higher than in the case of CBD. Moreover, in the presence of MβCD, the “retained” values are higher than those without the cyclodextrin, confirming the penetration enhancer activity of this excipient [68].

**Figure 6:**
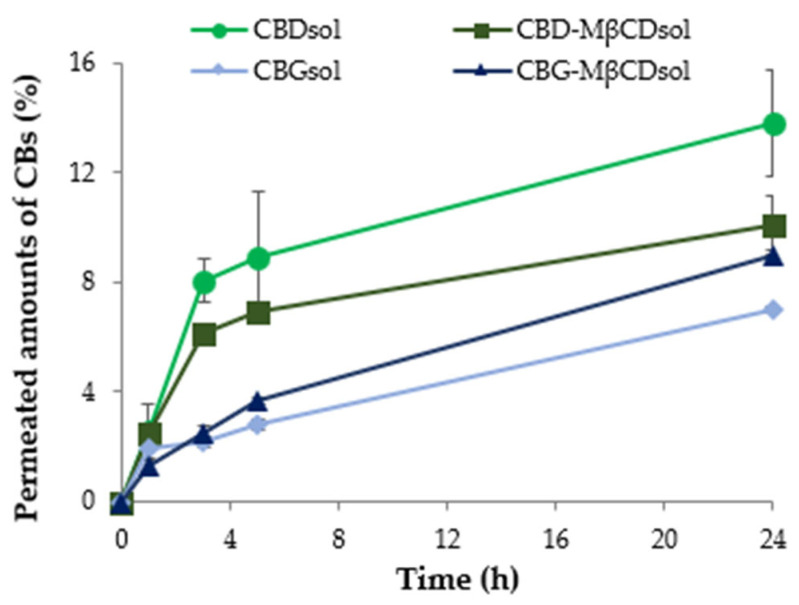
Permeation profiles of CBs from the solutions of CBs and CBs-MβCDsol. The reported values are calculated with respect to the loaded amounts of cannabinoids in the donor compartment at the beginning of the experiments.

The permeation curves of CB solutions, constructed with the percentage values of the drugs present in the aliquots withdrawn at 1, 3, 5, and 24 h from the receptor phase, are pictured in Figure 6.

After 1 h from the beginning of the experiment, about 2–3% of CBD and CBG permeated the membrane. Then, an increase in the percentages of permeated CBs occurs for all the solutions, in particular, in the case of CBDsol and CBD-MβCDsol, drug concentrations are higher (6% and 8%) compared to those of CBGsol and CBG-MβCDsol (about 3%). CBD-MβCDsol permeated amounts are always lower compared to the cannabinoid without the cyclodextrin (CBDsol), differently from solutions containing CBG. Therefore, unlike what was observed for CBG, cyclodextrin limits the ability of CBD to permeate through the skin biomimetic membrane and does not significantly enhance its penetration and retention in the membrane.

Overall, this permeation study proved that CBG possesses higher potential skin retention, and as such it is more suitable than CBD for topical administration intended for a local therapeutic effect. This result could be ascribed to the CBG molecule containing a terpenic chain similar to that of squalene, a terpenoid naturally present in the human sebum. Moreover, CBG showed higher antioxidant activity compared to CBD. As a consequence, CBG was selected for the successive formulation of innovative topical preparations.

### 3.4. Appearance, pH, Drug Content and Viscosity of Emulgels

**Figure 7:**
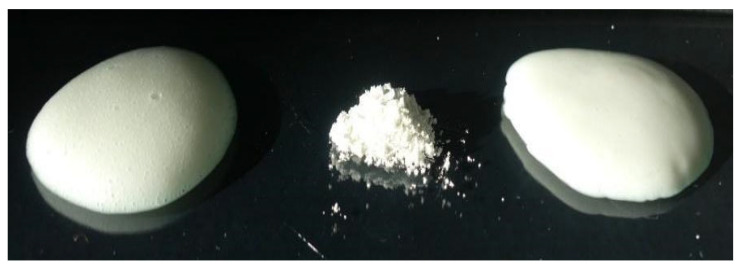
Starting emulgel (Ed), corresponding spray-dried powder (SDEd), and emulgel after reconstitution (Ed_R) (from left to right).

All the emulgels, Ea ÷ Ed and Ea_R ÷ Ed_R, were characterized by a white color, homogeneous appearance, and no particular odor. The reconstitution with water of powders obtained by the spray-drying of Ea ÷ Ed quickly led to the reconstitution of emulgels, which, compared to starting ones, were characterized by a brighter white color and an even more homogeneous appearance (Figure 7).

The pH of the emulgels was 5.86 ± 0.15 for Ea ÷ Ed and 6.44 ± 0.04 in the case of Ea_R ÷ Ed_R, similar to the physiological pH of the skin.

As expected, the experimental drug content of emulgels containing CBG was very close to the theoretical one, i.e., 1.12% for Ec and 1.05% for Ed (±0.02% for both).

**Figure 8:**
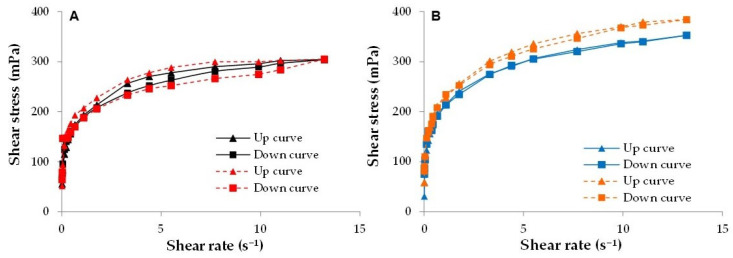
Up curves and down curves of (**A**) Ea (black-continuous lines) and Ea_R (red-dotted lines) and (**B**) Ed (blue-continuous lines) and Ed_R (orange-dotted lines).

The viscosity of all the emulgels at 20 rpm was in the range of 5900 ÷ 7100 mPa·s (spindle 25; 33.0 ± 0.5 °C). In the presence of CBG and MβCD, only a slight increase in the consistency of the emulgels occurs. In fact, at 20 rpm, the viscosity values of Ed and Ed_R were 6650 ± 20 mPa·s and 7150 ± 150 mPa·s, while Ea and Ea_R values were 5950 ± 250 mPa·s and 6000 ± 550 mPa·s. The flow curves reported in Figure 8 show that emulgels possess a shear-thinning behavior: their viscosity, which is the slope of the flow curve, gradually decreases under shear stress. As expected for non-Newtonian plastic fluids, all the curves present a yield value: the resistance to initial flow can be attributed to the polymeric network of the emulsifier, containing xanthan gum, tapioca starch and sodium alginate.

The up and down viscosity ramps of Ea and Ed (Figure 8, continuous lines) are almost superimposed, with no significant area of the hysteresis loop. On the contrary, the up and down flow curves of Ea_R and Ed_R (Figure 8, dotted lines) are slightly non-overlapping, indicating the thixotropic behavior of the reconstituted systems, which, however, is very weak and can be ascribed to a partial structuration of the polymeric network of the emulsifier in these emulgels [69]. Based on these results, the spray-drying process did not substantially affect the consistency of the formulations: reconstituted emulgels showed almost the same rheological behavior as the corresponding starting ones.

### 3.5. Spray-Drying Process Yield and Moisture Content

SDEa÷SDEd, obtained by converting 20 g of emulgels (Ea ÷ Ed) via spray-drying, appeared as fine white powders. All the powders were characterized by a low moisture content (determined by thermogravimetric analysis), and the percentage values decreased from almost 5% to 2% for SDEa to SDEd (Table 5). The percentage yield values of the process are included in a range between about 36% and 65%, which is satisfying for laboratory-scale production, even considering that the process was carried out with small quantities of samples.

It was observed that the higher the solid percentage in the formulations (Table 1), the higher the yield (Table 5). SDEd with 5% solids showed higher yield (65.0 ± 6.0%) compared to SDEa (36.0 ± 0.2%) with 3% solids. Therefore, the presence of cyclodextrin is fundamental for enhancing the process yield. SDEb showed a higher yield compared to its control SDEa; however, the increased yield obtained for SDEd compared to SDEb was not statistically significant. In addition, as expected, the cyclodextrin enhanced the efficiency of the drying process [56], providing a powder with a lower moisture content (3.8 ± 0.4% for SDEb). It is not surprising that the yield obtained from the spray-drying process of 100 g of Ea was higher, equal to 75.8 ± 2.2%; this result is even more significant given the low percentage of solids present in this formulation (just 3% of polymer-based emulsifier).

### 3.6. Drug Content, TGA, ATR-FTIR and XRPD of Powders

The experimental drug content of spray-dried powders, determined by HPLC to evaluate if CBG was stable throughout the spray-drying process, was 13.6 ± 1.1% in the case of Ec and 13.1 ± 0.2% for Ed, higher than approximately 80% and 90% of the theoretical drug content, i.e., 16.7% and 14.3%, respectively (Table 2).

**Figure 9:**
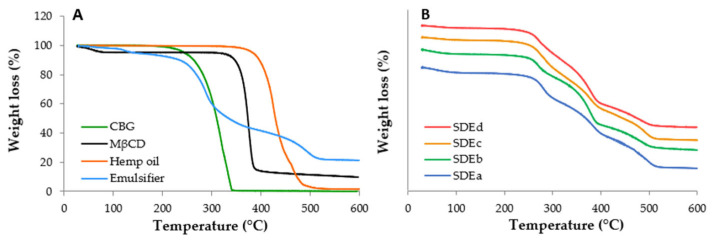
Thermogravimetric profiles of (**A**) single components of emulgels and (**B**) spray-dried powders.

The qualitative composition of the powders was also evaluated by thermogravimetric analysis (Figure 9). Spray-dried powders (Figure 9B) showed TGA profiles that were the result of the single components (Figure 9A), and the experimental weight loss percentages of the ingredients were comparable to the theoretical ones, suggesting that the spray-drying process did not induce significant modifications to the theoretical composition of the emulgels.

**Figure 10:**
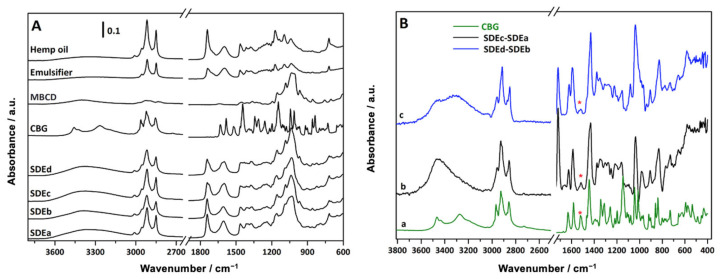
(**A**) ATR-FTIR spectra of single components of the emulgels and spray-dried powders (SDEa ÷ SDEd). (**B**) ATR-IR spectrum of CBG (a) and different spectra obtained after subtracting the SDEa spectrum from the SDEc spectrum (b) or the SDEb spectrum from the SDEd spectrum (c). The signal at 1518 cm^−1^ uniquely assigned to CBG is marked with an red asterisk (*).

In Figure 10A, the ATR-FTIR spectra of the single components of the formulations (CBG, MβCD, emulsifier, hemp oil) are reported along with the spectra of the spray-dried powders (SDEa ÷ SDEd). The spectra of both hemp oil and the emulsifier present intense C-H stretching vibration signals in the high-frequencies region and a complex set of signals in the low-frequencies region, reflecting the complex nature of the medium, and are dominated by intense C=O stretching absorption at about 1740 cm^−1^. Although the signals due to the presence of MβCD, hemp oil, and emulsifier are quite easily identified in the powders’ spectra, to evidence signals uniquely ascribable to the CBG molecule, SDEa and SDEb (with MβCD) spectra were subtracted from the SDEc (with CBG) and SDEd (with CBG and MβCD) spectra, respectively. After subtraction (Figure 10B), the signal at 1518 cm^−1^ arises due to ring stretching modes of CBG.

**Figure 11:**
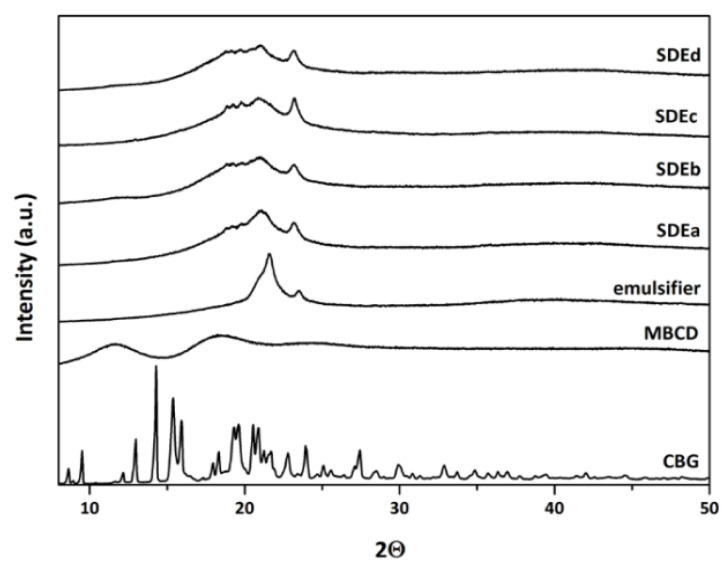
XRPD patterns of single components of emulgels and spray-dried powders.

The XRPD patterns of the spray-dried powders have been compared to the XRPD patterns of the single components of the formulations (Figure 11). Pure CBG shows several sharp reflections at 2Θ of 8.63°, 9.51°, 12.16°, 12.98°, 14.29°, 14.29°, 15.39°, 15.92°, 19.31°, 19.62°, 20.55°, 20.90°, 23.93°, and 27.42°; the emulsifier is characterized by two components in the 20–24 2Θ range, and MβCD shows three broad components in the 10–30 2Θ range due to its amorphous nature [61]. As observed also for TGA and ATR-FTIR analyses, the XRPD patterns of the spray-dried powders appear as the superimposition of the patterns of the single components of the formulation, with the only exception that characteristic reflections of CBG are no longer visible; this can be attributed to the amorphous state of the molecule within the formulation, confirming spray-drying as a useful technique for manufacturing amorphous solid materials.

### 3.7. Dynamic Angle of Repose and Particle Size Distribution of Powders

The flowability of powders was not satisfying, but it can be noticed that the simultaneous presence of CBG and MβCD slightly enhanced the powder flow. In fact, the angle of repose for SDEa was 58.6 ± 2.3 degrees (very poor flow property), and for SDEd, it was 48.0 ± 3.5 degrees (poor flow property), while those of SDEb and SDEc were not measurable. These powders, in fact, exhibited a significant tendency to stick to the walls of the drum.

The granulometric analysis reveals that the particle dimensions of SDEa and SDEd are statistically different: D90 126.7 ± 0.3 μm and 108.3 ± 0.2 μm, respectively (SPAN 4.9 ± 0.1). Nonetheless, this result is not to be considered relevant for re-dispersible powders intended for cutaneous administration.

Since the ability of powders to flow is fundamental in the pharmaceutical industry [70], the flowability of the spray-dried powders must be improved for future developments, and further investigations are needed to facilitate their handling; for this purpose, suitable fillers and excipients, such as polyols, can be added to the formulations.

## 4. Conclusions

At the time of writing, CBG-based solid formulations have yet to be proposed. In this work, the spray-drying technique allowed for obtaining re-dispersible powders containing CBG for the preparation of extemporaneous emulgels. These powders could represent a new metered-dosage form for the localized treatment of cutaneous afflictions, such as acne and psoriasis.

Further investigation would focus on the in vitro assessment of the efficacy of CBG formulations, and release studies will be performed using biological membranes, like human skin from donors. In addition, the spray-drying yield could be enhanced, along with the identification of optimized parameters for the scalability of the process.

## Figures and Tables

**Table 1:** Percentage composition of the emulgels (Ea ÷ Ed).

Formulation	Composition (% *w*/*w*)			
*Emulgels*	*Ea*	*Eb*	*Ec*	*Ed*
Hemp oil	2.0	2.0	2.0	2.0
Emulsifier and gelling agent	3.0	3.0	3.0	3.0
MβCD	-	1.0	-	1.0
CBG	-	-	1.0	1.0
Water	95.0	94.0	94.0	93.0

**Table 2:** Percentage theoretical composition of the powders (SDEa ÷ SDEd).

Formulation	Theoretical Composition (% *w*/*w*)			
*Spray-Dried Powders*	*SDEa*	*SDEb*	*SDEc*	*SDEd*
Hemp oil	40.0	33.3	33.3	28.6
Emulsifier and gelling agent	60.0	50.0	50.0	42.8
MβCD	-	16.7	-	14.3
CBG	-	-	16.7	14.3

**Table 3:** T onset and (theoretical and experimental) weight loss percentages of cannabinoids, MβCD, pm, and kn systems (temperature range 125 ÷ 500 °C).

Sample	T Onset (° C)	Theoretical Weight Loss (%)	Experimental Weight Loss (%)
CBD	140.8	100.0	99.8
pmCBD	143.4	50.0	53.0
knCBD	186.6	50.0	40.8
CBG	135.7	100.0	100.0
pmCBG	152.4	50.0	51.0
knCBG	170.7	50.0	40.2
MβCD	310.0	100.0	85.5

**Table 4:** Distribution of cannabinoids, expressed as relative percentages, at the end of the permeation tests. The percentages of the “retained”, “washed”, “stripped” and “permeated” fractions refer to the “Total diffused” amount. Values with the same letters in the same row are not significantly different.

CBs	CBDsol (%)	CBD-MβCDsol (%)	CBGsol (%)	CBG-MβCDsol (%)
“In donor phase”	47.3 ± 3.3 ^a^	49.2 ± 2.5 ^a^	53.1 ± 1.2 ^a^	47.5 ± 4.6 ^a^
“Total diffused”	21.9 ± 0.6 ^A^	20.5 ± 0.6 ^A^	23.9 ± 2.4 ^A^	35.1 ± 4.7 ^B^
“Retained”	34.1 ± 1.1	48.7 ± 0.2	67.1 ± 7.4	69.6 ± 7.0
“Washed”	2.8 ± 2.0	1.1 ± 1.2	4.6 ± 2.5	0.7 ± 0.8
“Stripped”	0.3 ± 0.1	0.5 ± 0.2	0.2 ± 0.1	0.2 ± 0.1
“Permeated”	63.0 ± 2.6	49.7 ± 11.2	29.4 ± 4.1	28.6 ± 7.6

**Table 5:** Percentage yield and moisture content of the spray-dried powders. For each column, different letters correspond to significant differences between groups.

Spray-Dried Powders	Yields (%)	Moisture Content (%)
SDEa	36.0 ± 0.2 ^b^	5.3 ± 0.7 ^a^
SDEb	54.0 ± 4.0 ^a^	3.8 ± 0.4 ^b^
SDEc	50.7 ± 6.3 ^ab^	2.6 ± 0.3 ^c^
SDEd	65.0 ± 6.0 ^a^	2.0 ± 0.2 ^c^

## Data Availability

The data presented in this study are contained within the Article and the Supplementary Materials file.

## Supplementary Materials

The following supporting information can be downloaded at: https://www.mdpi.com/article/10.3390/pharmaceutics15122747/s1, Figure S1: 1H NMR of CBD in CDCl3, 400 MHz; Figure S2: 1H NMR of CBG in CDCl3, 400 MHz.
