# Supplementary material for: Spray-Dried Powder Containing Cannabigerol: A New Extemporaneous Emulgel for Topical Administration
Source: Pharmaceutics. 2023 Dec 8;15(12):2747. doi: 10.3390/pharmaceutics15122747 (PMC10747370; doi:10.3390/pharmaceutics15122747)
Supplement: Supplementary file 1 [file pharmaceutics-15-02747-s001.zip › pharmaceutics-2735454-supplementary.pdf]

Development of a Spray-Dried Topical Formulation Containing Cannabigerol

Alice Picco [1], Lorena Segale [1,2], Ivana Miletto [1], Federica Pollastro [1], Silvio Aprile [1], Monica Locatelli [1], Elia Bari [1], Maria Luisa Torre [1] and Lorella Giovannelli [1,2]

Supporting information:

Figure S1. ^{1}H NMR of CBD (**A**) in $CDCl_3$, 400 MHz

Figure S2. ^{1}H NMR of CBG (**B**) in $CDCl_3$, 400 MHz

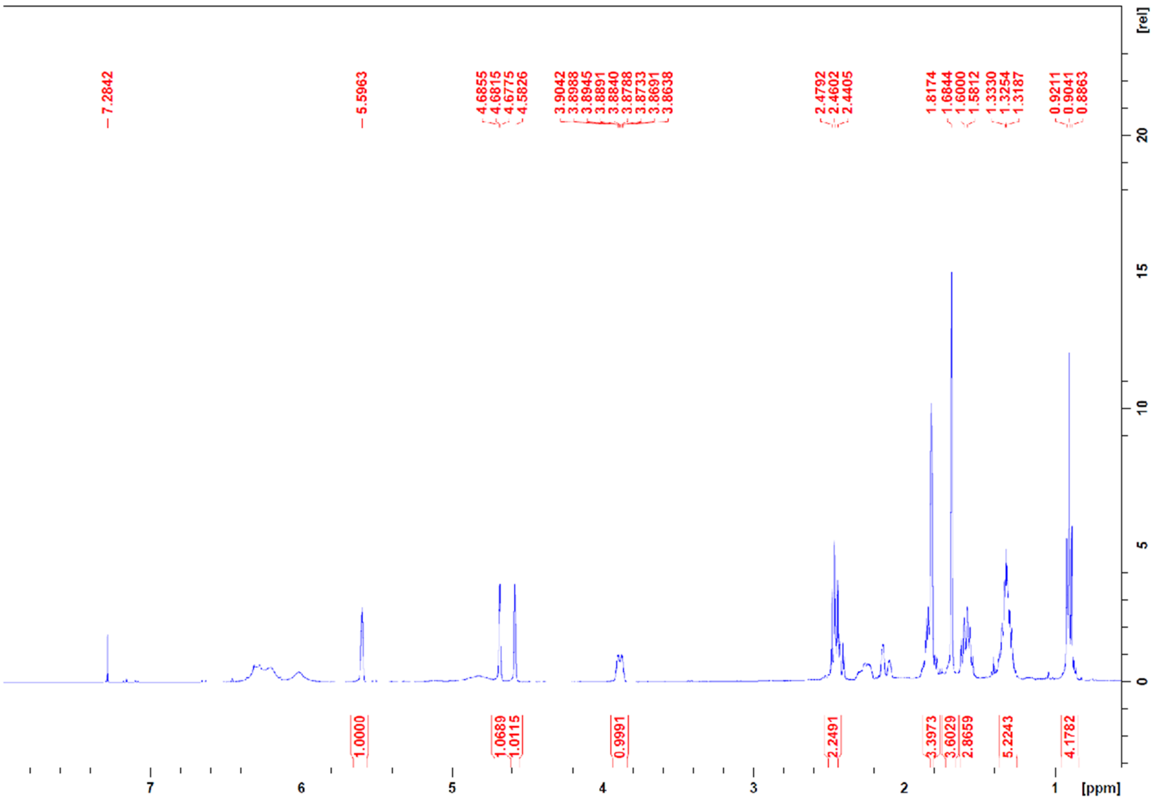

Figure S1. ^{1}H NMR of CBD (**A**) in $CDCl_3$, 400 MHz.

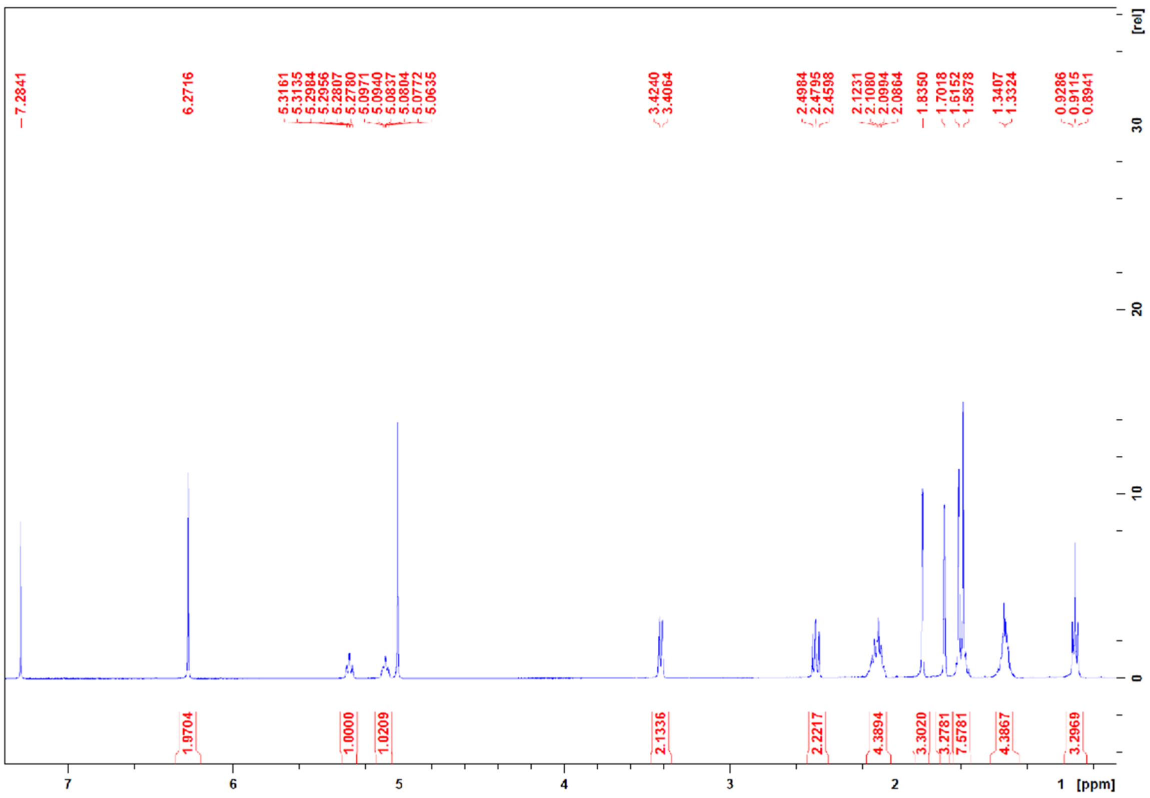

Figure S2. ^{1}H NMR of CBG (**B**) in $CDCl_3$, 400 MHz.
